# The development of visuospatial abilities and their impact on laparoscopic skill acquisition: a clinical longitudinal study

**DOI:** 10.1007/s00464-022-09328-1

**Published:** 2022-05-31

**Authors:** Tina Vajsbaher, Holger Schultheis, Sonja Janssen, Dirk Weyhe, Hüseyin Bektas, Verena Uslar, Nader Francis

**Affiliations:** 1 Bremen Spatial Cognition Center, University of Bremen, Enrique-Schmidt-Straße 5, 28359 Bremen, Germany; 2 Faculty for Human and Health Sciences, University of Bremen, Bremen, Germany; 3 Institute for Artificial Intelligence, University of Bremen, Bremen, Germany; 4 University Hospital for Visceral Surgery, Pius-Hospital Oldenburg, Carl Von Ossietzky University Oldenburg, Oldenburg, Germany; 5 Department for General, Visceral-and-Surgical Oncology, Klinikum Bremen-Mitte, Germany; 6 Division of Surgery and Interventional Science, University College London, London, UK; 7 The Griffin Institute, Northwick Park and St Mark’s Hospital, Y Block, Watford Road, Harrow, HA1 3UJ Middlesex UK

**Keywords:** Visuospatial Abilities, Laparoscopic skills, Longitudinal

## Abstract

**Objectives:**

To investigate how visuospatial abilities develop and influence intraoperative laparoscopic performance during surgical residency training programmes.

**Background:**

Laparoscopic surgery is a challenging technique to acquire and master. Visuospatial ability is an important attribute but most prior research have predominantly explored the influence of visuospatial abilities in lab-based settings and/or among inexperienced surgeons. Little is known about the impact of visuospatial profiles on actual laparoscopic performance and its role in shaping competency.

**Method:**

A longitudinal observational cohort study using a pair-matched design over 27 months. At baseline, visuospatial profiles of 43 laparoscopic surgeons of all expertise levels and 19 control subjects were compared. The development of visuospatial abilities and their association with intraoperative performance of 18 residency surgeons were monitored during the course of their laparoscopic training.

**Results:**

Laparoscopic surgeons significantly outperformed the control group on the measure of spatial visualisation (*U* = 273.0, *p* = 0.03, η2 = 0.3). Spatial visualisation was found to be a significant predictor of laparoscopic expertise (R^2^ = 0.70, F (1.60) = 6.788, *p* = 0.01) and improved with laparoscopic training (B = 4.01, *SE* = 1.83, *p* = 0.02, 95% CI [0.40, 7.63]). From month 6 to 18, a strong positive correlation between spatial visualisation and intraoperative depth perception (*r* = 0.67, *p* < 0.01), bimanual dexterity (*r* = 0.60, *p* < 0.01), autonomy (*r* = 0.78, *p* < 0.01) and the total score (*r* = 0.70, *p* < 0.01) were observed but a strong relationship remained only with autonomy (*r* = 0.89, *p* < 0.01) and total score (*r* = 0.80, *p* < 0.01) at 18 months.

**Conclusion:**

In this longitudinal cohort study, visuospatial abilities associate with laparoscopic skills and improve with training. Spatial visualisation may be characteristic of laparoscopic expertise as it has clear association with competency development during laparoscopy residency training programme.

**Supplementary Information:**

The online version contains supplementary material available at 10.1007/s00464-022-09328-1.

Visuospatial complexities associated with performing and mastering laparoscopy are well documented in the literature [1, 2]. Facets such as lack of depth perception, 2D visualisation of 3D anatomy, eye-hand coordination and counterintuitive dexterous movements (i.e. Fulcrum effect) all contribute towards the perceived difficulty of mastering the procedure [3, 4]. Visuospatial ability refers to mental comprehension and conceptualization of visual representations and spatial relationships within a perceptual scene [5]. The focus on spatial cognition was prompted by research findings reporting a positive correlation between visuospatial abilities and laparoscopic performance and outcomes [6]. That is, visuospatial abilities have been linked with better performance, shorter operative times and better patient outcomes [7]. Yet, the true extent to which visuospatial abilities influence laparoscopic performance and impact skill acquisition remains largely unclear. Whereas some studies found visuospatial abilities such as mental rotation and spatial visualisation to show an enduring influence over laparoscopic skill acquisition [8, 9], others found the influence of the same abilities to diminish with practice over time [10, 11]. Additionally, the existing evidence on which we currently base our understanding comes from studies exploring the role of visuospatial abilities in laypeople or medical students on surgical simulators [7]. Whether visuospatial abilities develop with advanced laparoscopic training so that expert laparoscopic surgeons possess superior visuospatial abilities remains to be answered. Additionally, it is unclear how these abilities influence the actual intraoperative laparoscopic performance during residency training programmes. This current study aimed to clarify these divergent findings by determining the visuospatial profiles of laparoscopic surgeons across all experience levels and longitudinally exploring how visuospatial abilities develop and or influence laparoscopic skill acquisition during the surgical residency training programme.

## Method

### Design

A longitudinal observational cohort study using a pair-matched design over 27 months. Control subjects were pair-matched with residency surgeons based on visuospatial profiles, and to the extent possible, gender. The longitudinal data collection began in January 2018 and ended in March 2020. All subjects were recruited following the convenience sampling technique structured around voluntary participation. Informed consent was obtained prior to the study. The study was ethically approved by the Department of Human and Health Sciences at the University of Bremen, Germany.

### Participants

A total of 62 subjects were recruited: 43 (69.4%) surgeons specialised or training in laparoscopy and 19 (30.6%) control subjects. The sample consisted of 26 (41.9%) residency surgeons, 17 (27.4%) senior surgeons and 19 (30.6%) control subjects. At baseline, visuospatial profiles of all subjects were quantitatively compared (Table 1).

**Table 1:** Demographic description of the participants

Baseline	Senior surgeons	Residency surgeons	Control subjects
	*n *= 17	*n *= 26	*n *= 19
Gender			
Female	4	13	12
Male	13	13	7
Age			
Mean (SD)	48 (3)	33 (4.2)	25 (3.1)
Range	39–57	26–37	21–37

Longitudinal analysis		Residency surgeons	Control Subjects
		*n *= 18	*n *= 18
Gender			
Female		6	11
Male		12	7
Age			
Mean (SD)		31 (2.28)	25 (3.1)
Range		30–37	21–37

The surgeon cohort included all surgical staff from two departments for general and visceral surgery at Klinikum Bremen-Mitte and Pius Hospital Oldenburg. Among the residents were seven first and second-year trainees with no prior laparoscopic experience, ten junior residents in their third and fourth training year and nine senior residents in their final fifth and sixth training year. The senior surgeon cohort included two surgical specialists, 12 senior consultants and three clinical directors (i.e. chief surgeons). Resident surgeons reported having an average of 4.8 (*SD* = 2) years of laparoscopic experience with senior surgeons reporting an average of 18 years (*SD* = 11). From the sample included in the baseline, 36 subjects participated in the longitudinal analysis: 18 residency surgeons and 18 control subjects. Among the residents were ten junior trainees (55.5%) in their third and fourth residency year and eight senior trainees (44.5%) in their final fifth and sixth training year. The control subjects were recruited from the general population or the University of Bremen. The students were either bachelor and master university students studying computer science, psychology, and/or public health. See Table 1 for the descriptive overview of the participant’s demographic at both baseline and longitudinal level.

#### Inclusion and exclusion criteria

For visuospatial testing, all surgical staff at all seniority and training levels from the two departments for general and visceral surgery were included. For the longitudinal analysis, the trainees in their formal residency programme undergoing surgical training in laparoscopy surgery were included. In Germany, the first two years of residency training are devoted to basic clinical training and rotations (six months in emergency care, six months in intensive care and one year in surgical department) followed by a 4-year surgical specialisation training (i.e. general surgery) (see Drossard [12] for the overview of the German residency programme). Seven residents in basic training were therefore excluded from the longitudinal clinical analysis. For the control subjects, the inclusion criteria called for any healthy individual with no previous experience in cognitive psychometric testing and no history of cognitive or neuropsychological impairment.

### Instruments and materials

Four validated visuospatial psychometric tests were used in this study. This set(s) of tests have been previously found to predict laparoscopic technical performance [7]. The aptitude tests used were (1) The Perspective Taking/ Spatial Orientation Test (PTSOT) [13], (2) A modified Guay’s Visualization of Views Test (GVVT) [14], (3) A Mental Rotation Test (MRT-A) [15], and (4) The Pictorial Surface Orientation (PicSOr) [16]. See appendix A for further information on the visuospatial tests.

Laparoscopic performance of residency surgeons was assessed using the Global Operative Assessment of Laparoscopic Skills (GOALS) [17]. The assessment tool includes a five-item global rating scale with a ten-item checklist measuring depth perception, bimanual dexterity, efficiency, tissue handling and autonomy [17]. Performance is measured out 25 points. For each item, a score of one describes poor performance and a score of five excellent performance. Trainees intraoperative performance was evaluated by the respective senior surgeon responsible for their training and supervising the case at hand. All senior surgeons received a briefing on the nature of the assessment tool prior to the study. Additionally, a three-point case difficulty scale (1—easy, 2—challenging, 3—difficult) based on senior surgeons’ subjective perception of the surgical complexity (i.e. type of the intervention, anatomical variations, inflammation or complications). A five-point American Society of Anaesthesiologists (ASA) scale was also included to control for any confounding effects of the patient commodities that could contribute towards the complexity of the surgical case (i.e. obesity or poor condition).

### Data collection and data analysis

First, all 62 subjects completed the four visuospatial tests under the same set of instructions and testing conditions. In the scope of the longitudinal analysis, visuospatial testing was undertaken after every tenth laparoscopic case completed by the resident surgeon as the main operator or first assistant. This served as an additional measure to reduce the re-testing effect (i.e. improvement in performance due to the over-memorisation of the task). Control subjects were tested in the same timeframe as their matched residents.

Descriptive statistics for the visuospatial scores are reported using the median and the interquartile range (IQR). At baseline, the quantitative comparisons of visuospatial profiles between surgeons and control group was computed using the Mann–Whitney *U* test. Visuospatial comparison between expert surgeons, residents and control group was computed using the Kruskal–Wallis *H* test. A linear regression was conducted to explore whether a surgeon's seniority level can predict visuospatial profiles of surgeons while controlling for confounding factors such as gender, age and years of laparoscopic experience. The longitudinal data exploring the development of visuospatial abilities over the 27 months was analysed using the multilevel growth curve modelling, a method described by Shek & Ma [18]. Group (residents vs control) was treated as a time-invariant covariate and group x time interaction was included in the models. The impact of visuospatial abilities on intraoperative performance was analysed using longitudinal repeated measures correlation. First, within-subject correlation was conducted to explore whether an increase in visuospatial abilities leads to an increase in intraoperative score within an individual over time. This was computed using repeated measures correlation coefficient ‘rmcorr’ function on the R-statistics statistical programming software [19]. Second, the between-subjects repeated correlation was computed to explore the individual differences by exploring whether surgeons with higher visuospatial abilities also tended to have higher intraoperative scores. This was computed using weighted Pearson’s correlation between subjects mean aptitude score and mean intraoperative score [20].

## Results

### Section 1: baseline exploration of visuospatial profiles

#### Do laparoscopic surgeons possess better visuospatial abilities than control subjects?

Laparoscopic surgeons had a notably better performance on GVVT than control subjects (surgeons M = 20.50 vs control M = 11.10). Surgeons also showed better performance on perceptual-motor skills as measured by PicSOr (surgeons M = 0.50 vs control M = 0.26). Both surgeons and control subjects showed similar average performance on mental rotation measured by MRT-A (surgeons M = 10 vs control M = 11). A Mann–Whitney *U* test revealed a significant group difference with a medium effect size on the GVVT measure (*U* = 273.0, *p* = 0.03, η2 = 0.3), with surgeons significantly outperforming the control group. No significant group differences were observed on measures of PTOST (*U* = 328.0, *p* = 0.22, η2 = 0.1), the MRT (*U* = 291.5, *p* = 0.79, η2 = 0.1) or the PicSOr *(U* = 297.0, *p* = 0.09, η2 = 0.3) measures were found.

#### Do expert surgeons possess better visuospatial abilities than residents and control group?

Expert laparoscopic surgeons showed notably better performance on the GVVT (M = 20.25) than did residency surgeons (M = 15.65) and control subjects (M = 11.10). On the PicSOr, residency surgeons showed a slightly better performance compared to both expert and control subjects (residents M = 0.55 vs. expert surgeons M = 0.45 vs. control M = 0.26). No notable group differences were observed on the MRT-A. Kruskal–Wallis H test revealed no statistical group differences between residents, expert surgeons and control subjects on any of the four visuospatial measures were observed, as seen in Table 2. Expert surgeons group rank on the GVVT measure (Mean Rank = 37.62) was higher than those of residency surgeons (Mean Rank = 30.48) and control subjects (Mean Rank = 24.68) Table 2.

**Table 2:** Kruskal–Wallis H test measuring cohort differences in visuospatial aptitudes

Measure	Group	N	Rank	*H*	*X*^*2*^	*p*	η^2^
PTSOT	Residents	26	32.77	0.95	1.99	0.37	0.02
	Experts	17	33.29				
	Control group	19	28.16				
GVVT	Residents	26	30.48	4.75	3.72	0.06	0.05
	Experts	17	37.62				
	Control group	19	24.68				
MRT-A	Residents	26	33.00	0.32	1.61	0.45	0.03
	Experts	17	30.21				
	Control group	19	30.61				
PicSOr	Residents	26	33.75	2.76	4.02	0.13	0.01
	Experts	17	34.44				
	Control group	19	25.75				

*p* < .05 level of significance. *N *= number of participants, *H* = Kruskal–Wallis value, *X*^*2* =^ Chi-square value, *p* = *p*-value, η^*2*^ = Eta squared effect size

#### Does visuospatial aptitude level correlate with the years of laparoscopic experience among surgeons?

A linear regression explored whether years of laparoscopic experience can predict visuospatial performance of surgeons. The results revealed that years of experience predicted 70% of performance variance on the GVVT measure (R^2^ = 0.70, F (1,60) = 6.788, *p* = 0.01). The score on the GVVT increased by 0.34 point with every year of experience (Beta = 0.34, SE = 0.128, 0.01, 95% CI [0.8, 0.29]). Experience in laparoscopy was not a significant predictor of surgeon’s performance on the PTSOT (Beta =  − 0.16, SE = 0.05, *p* = 0 0.48, 95% CI [− 0.06, 0.12]), MRT-A (Beta =  − 0.25, SE = 0.24 *p* = 0.40, 95% CI [− 0.67, 0.27]) and PicSOr (Beta = 3.40, SE = 2.88, *p* = 0.24, 95% CI [− 2.34, 2.18]).

### Section 2: longitudinal analysis of skill development

#### Longitudinal data overview

From the 36 recruited residents’ subjects at the baseline, 32 subjects were tracked over the 27 months. Four subjects dropped out of the study in the course of 27 months: Three residents left the respective clinics and one control subjects relocated to another country. Data from two residents were lost between the 6^th^ and 24^th^ month due to rotations in another clinic. The most significant loss of data occurred between month 24 and 27 due to the COVID-19-related national restrictions and cancellation of elective surgeries. Complete data from 12 residents and 17 control subjects were collected at that time. The median follow-up period was 15 months.

#### Laparoscopic procedures assessed

A total of 164 baseline visuospatial measures were collected and 603 intraoperative assessments among over the 27 months. The intraoperative assessments included 255 (42.1%) laparoscopic cholecystectomies (CHE), 202 (33.4%) totally extra-peritoneal (TEP), 66 (10.9%) diagnostic/exploratory laparoscopies (DI/EX), 59 (9.4%) appendectomies (APP), 12 (2%) intraperitoneal onlay mesh (IPOM), seven (1.2%) sigmoid resections (SR) and two other (1%) individual laparoscopic procedures (i.e. nephrectomy and lymphadenectomy). The average case difficulty was rated as ‘medium’ with the average case ASA scale of 2 (i.e. a mild systemic disease). The average score across for each five of the intraoperative items measured by the GOALS was 3 (*IQR* = 1) (i.e. good performance) with the average total score over time being 15 (*IQR* = 4.0) out of 25. Seven (1.2%) laparoscopic cases were converted due to obstruction of visualisation caused by inflammation or bleeding.

#### Visuospatial development over time

**Fig. 1:**
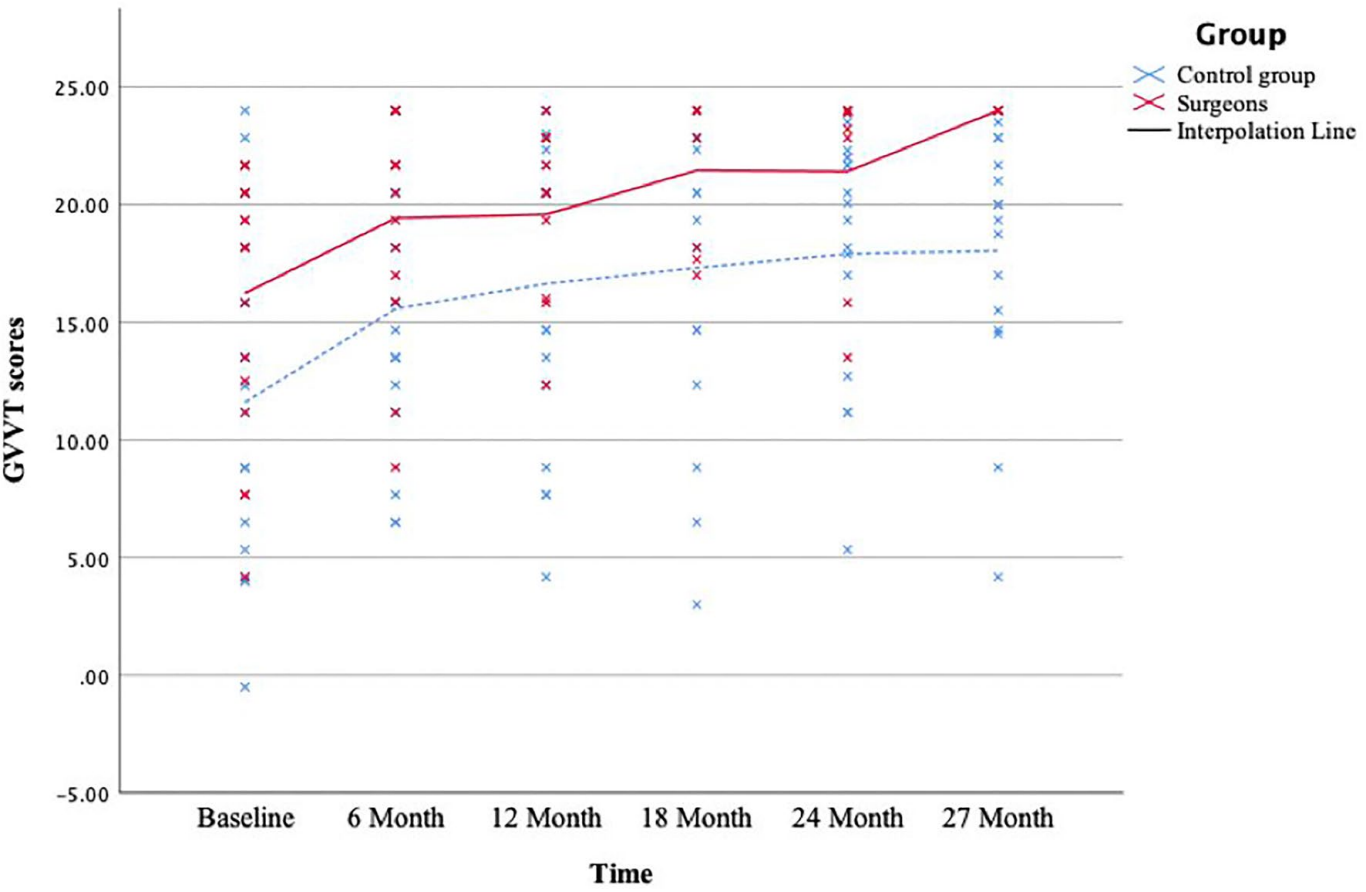
A graphical illustration of the spatial visualisation trajectory per cohort group over the 27 months

Linear mixed-effect modelling explored whether the group (residency surgeons or control group) can predict the shape of individual aptitude trajectories over time. All subjects showed a significant improvement in their visuospatial abilities over the 27 months (all *p* < 0.02). Table 3 illustrates the descriptive overview of the aptitude scores per residency surgeons and control subjects across each time condition. Across all aptitude tests, the group was a significant predictor of individual trajectories for spatial visualisation measured by the GVVT only (*p* < 0.01). Residency surgeons showed a significantly faster linear rate of score increase (B = 4.01, *SE* = 1.83, *p* = 0.02, 95% CI [0.40, 7.63]) than did control subjects. That is, whereas residency surgeons started with higher performance and continued to show improvement on spatial visualisation in the scope of laparoscopic training, the rate of improvement in control subjects plateaued over time, as illustrated in Fig. 1. At the end of the 27 months, residency surgeons reached the maximum performance on the GVVT measure. Group factor was not a significant predictor of individual trajectories over time on the measures of PTSOT (B = 2.37, *SE* = 3.24, *p* = 0.89, 95% CI [− 1.75, 0.22]), MRT-A (B = 0.49, *SE* = 1.25, *p* = 0.70, 95% CI [− 2.00, 3.97]) and PicSOr (B = 0.03, *SE* = 0.04, *p* = 0.49, 95% CI [− 0.05, 0.11]). See appendix B for the graphical illustration of all aptitude trajectories per cohort group.

**Table 3:** Descriptive summary of aptitude score for both groups across all time conditions

	Resident surgeons				Control group			
	PTSOT	GVVT	MRT-A	PicSOr	PTSOT	GVVT	MRT-A	PicSOr
	Median (IQR)	Median (IQR)	Median (IQR)	Median (IQR)	Median (IQR)	Median (IQR)	Median (IQR)	Median (IQR)
Baseline	71.73 (17.63)	18.17 (8.33)	9.50 (7.50)	.57 (.59)	64.15 (33.09)	11.73 (11.38)	11.00 (4.25)	.28 (.66)
6th month	79.58 (15.61)	21.09 (7.28)	14.50 (7.75)	.55 (.42)	67.07 (17.79)	15.25 (8.46)	13.50 (7.75)	.25 (.47)
% score change	+ 10.9%	+ 16.2%	+ 52. 6%	− 3.5%	+ 4.6%	+ 30.1%	+ 22.7%	− 10.7%
12^th^ month	79.61 (13.09)	22.50 (6.87)	14.50 (3.75)	.67 (.50)	71.10 (26.23)	20.50 (12.00)	15.00 (8.00)	.45 (.38)
% score change	+ 11.0%	+ 28. 3%	+ 52.6%	+ 17.5%	+ 10.1%	+ 74.8%	+ 36.4%	+ 60.7%
18^th^ month	81.50 (23.92)	23.42 (6.20)	15.00 (6.75)	.93 (.29)	77.83 (15.32)	19.33 (9.33)	17.00 (8.50)	.55 (.65)
% score change	+ 13.6%	+ 28.9%	+ 57.9%	+ 63.2%	+ 21.3%	+ 67.8%	+ 54.6%	+ 96.4%
24^th^ month	81.28 (8.97)	23.55 (6.42)	17.50 (5.00)	.96 (.59)	75.37 (22.14)	19.69 (8.14)	17.50 (8.75)	.54 (.77)
% score change	+ 13.3%	+ 29.6%	+ 84.2%	+ 68.4%	+ 17.5%	+ 67.9%	+ 59.1%	+ 92.9%
27^th^ month	86.31 (7.49)	24.00 (0.00)	19.00 (8.75)	.84 (.87)	69.73 (18.23)	19.67 (7.66)	18.50 (6.75)	.91 (.68)
% score change	+ 20.3%	+ 32.1%	+ 100%	+ 47.4%	+ 8.7%	+ 67.7%	+ 68.2%	+ 105.9%
Average change from the baseline (%)	+ 13.8%	+ 27.0%	+ 58.9%	+ 38.6%	+ 12.4%	+ 61.7%	+ 48.2%	+ 68.9%

#### Within-subjects correlation between visuospatial abilities and intraoperative performance over time

**Fig. 2:**
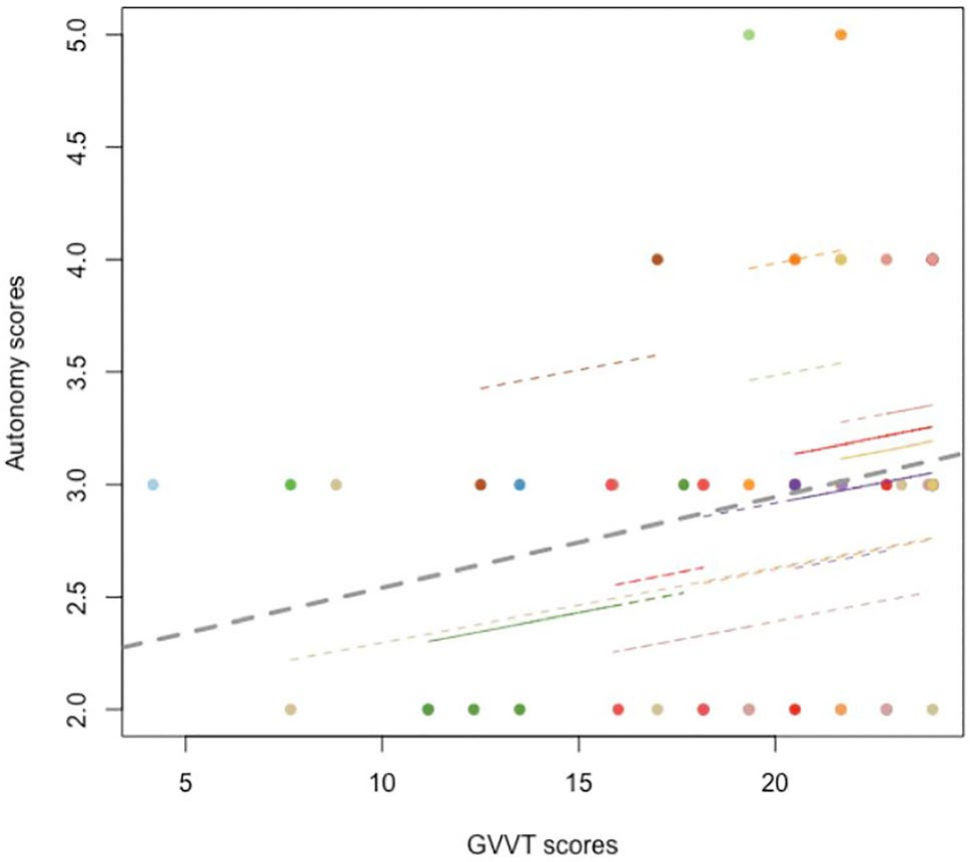
The Rmcorr plot illustrating a positive within-subject association between spatial visualisation and intraoperative autonomy. Colour lines representing slopes for each individuals. The dotted lines represents a common regression slopes across individuals

A significant positive correlation was observed between the GVVT and operative autonomy, as assessed by GOALS scores (rm = 0.35, *p* < 0.05) only, indicating that an increase in an individual’s spatial visualisation score was positively associated with the individuals increase in operative autonomy over time, as illustrated in Fig. 2. The correlation between other visuospatial measures and intraoperative items revealed predominantly small to medium effect sizes (effect size range: − 0.21 to 0.28), revealing these relationships varied extensively between subjects, as is seen in Table 4.

**Table 4:** Rmcorr correlation coefficient between visuospatial abilities and intraoperative skills

df = 43	Depth perception	Bimanual dexterity	Efficiency	Tissue handling	Autonomy	Total score
PTSOT						
*r*_rm_	− 0.13	− 0.07	− 0.09	0.07	− 0.21	− 0.09
*p* value	0.38	0.67	0.55	0.66	0.18	0.55
95% CI	− 0.28, 0.10	− 0.12, 0.32	− 0.19, 0.17	− 0.33, 0.20	− 0.29, 0.08	− 0.25, 0.14
GVVT						
*r*_rm_	0.15	0.03	0.08	− 0.07	0.35	0.08
*p* value	0.34	0.86	0.60	0.42	0.05*	0.59
95% CI	0.03, 0.51	0.06, 0.48	0.05, 0.50	− 0.24, 0.10	0.12, 0.49	0.02, 0.54
MRT-A						
*r*_rm_	0.14	0.09	0.17	0.03	0.28	0.17
*p* value	0.35	0.58	0.26	0.85	0.08	0.28
95% CI	− 0.20, 0.30	− 0.16, 0.27	− 0.20, 0.29	− 0.21, 0.33	− 0.03, 0.43	− 0.10, 0.36
PicSOr						
* r*_rm_	− 0.08	0.14	0.17	− 0.00	0.26	0.17
*p* value	0.58	0.37	0.27	0.99	0.09	0.28
95% CI	− 0.19, 0.27	− 0.02, 0.46	− 0.16, 0.22	− 0.21, 0.19	0.13, 0.50	− 0.02, 0.37

#### Time-aggregated between-subject correlation on the average visuospatial scores and intraoperative scores over time

All intraoperative assessments were time-aggregated following the same data collection period as visuospatial tests: 6th month, 12th month, 18th month and the 24th month. No correlations for month 27 were computed due to the small sample size following the COVID lockdown (*n *= 4). From the baseline to month 6, subjects with higher GVVT score had a significantly higher intraoperative scores on depth perception (*r* = 0.61, *p* < 0.01), bimanual dexterity (*r* = 0.59, *p* < 0.01) and autonomy (*r* = 0.65, *p* < 0.01). A moderate relationship between MRT and autonomy was observed (*r* = 0.45, *p* < 0.01) at month six and at month 12 (*r* = 0.70, *p* < 0.01). From month 12 to 18, only a strong relationship between GVVT and intraoperative items of depth perception (*r* = 0.74, *p* < 0.01), dexterity (*r* = 0.72, *p* < 0.01), autonomy (*r* = 0.86, *p* < 0.01) and total score (*r* = 0.74, *p* < 0.01) was observed. From month 18 to 24, only a strong relationship between GVVT and autonomy (*r* = 0.89, *p* < 0.01) and total score (*r* = 0.80, *p* < 0.01) remained (Table 5).

**Table 5:** Time-aggregated within-subject correlation between visuospatial abilities and intraoperative scores

Month 6 (*N *= 14)	Depth perception	Bimanual dexterity	Efficiency	Tissue handling	Autonomy	Total score
PTSOT	0.05	0.11	0.10	− 0.01	0.22	− 0.29
GVVT	0.61*	0.59*	0.02	0.48	0.65*	0.26
MRT-A	0.25	0.04	0.14	0.11	0.45*	0.12
PicSOr	0.42	0.24	0.30	0.23	0.45	0.11
Month 12 (*N *= 10)						
PTSOT	0.19	0.17	0.06	0.11	0.13	− 0.30
GVVT	0.67*	0.60*	0.58	0.56	0.78*	0.70*
MRT-A	0.41	0.33	0.39	0.41	0.70*	0.48
PicSOr	0.26	0.18	0.17	0.09	0.24	0.22
Month 18 (*N *= 8)						
PTSOT	− 0.10	− 0.20	− 0.07	0.03	0.04	− 0.37
GVVT	0.74*	0.72*	0.68	0.61	0.86*	0.74*
MRT-A	0.58	0.52	0.51	0.57	0.55	0.55
PicSOr	0.54	0.48	0.08	0.02	0.49	0.25
Month 24 (*N *= 8)						
PTSOT	0.34	− 0.27	− 0.24	0.12	− 0.30	− 0.37
GVVT	0.69	0.69	0.69	0.61	0.89*	0.80*
MRT-A	0.41	0.58	0.47	0.41	0.58	0.18
PicSOr	0.26	0.22	0.52	0.59	0.59	0.11

*Significant at *p* < 0.01

## Discussion

The results of this longitudinal study offer novel insights into the role of visuospatial abilities in promoting intraoperative performance and shaping laparoscopic training, competence and expertise in the technique. The findings revealed a clear pattern implicating spatial visualisation, that is mental inference of three-dimensional figures from a two-dimensional view, as characteristics of laparoscopic expertise and skill development. Spatial visualisation distinguished laparoscopic surgeons from control subjects, predicted years of laparoscopic experience and showed an enduring and strengthening association with laparoscopic skill development. In particular, spatial visualisation was found to improve in the context of laparoscopic training beyond mere re-testing and was found to promote operative autonomy over the course of residency training.

Although the combined results of this study are novel, our findings are in line with previous work by Risucci [21] and Keehner [23]. Risucci [21] tested visuospatial abilities of surgeons across all experience levels similarly reported surgeons outperforming control subjects on the measure of spatial visualisation. The enduring influence of spatial visualisation on simulated laparoscopic performance has also been previously reported by Keehner [23]. These complementary findings provide strong indication that spatial visualisation ability may be key to a successful laparoscopic performance and provide support to the claim that surgeons are largely trained and not born [24]. Additionally, the results from the longitudinal study revealed some interesting individual differences in laparoscopic skill development, demonstrating that different abilities are called upon at different stages of skill acquisition. The longitudinal inter-subjects correlation revealed that surgeons with higher mental rotation and perceptual-motor skills showed only an initial advantage over laparoscopic performance. Yet, as spatial visualisation improved in the context of laparoscopic training, these individual differences and their impact over skill development diminished. From the perspective of cognition, the results dispute the general claim from the skill acquisition theories [25], that the association between cognition and performance diminishes as skills become automatized. Whereas this was confirmed with mental rotation and perceptual-motor skills, the observed enduring influence of spatial visualisation provides yet another indication that the ability may be characteristic of laparoscopy.

These findings offer a valuable new insight into the underlying mechanism driving the disparate findings in the existing literature. Previous studies on the topic focused predominantly on assessing mental rotation abilities in largely naïve subjects with varying experience levels in laparoscopy at either a one-time measurement level or over a few weeks. The assumptions made by these studies that mental rotation abilities diminish with experience is supported [10, 11], yet, the generalised statement that the importance of visuospatial abilities diminishes with experience levels is refuted. As was clearly observed in this study, spatial visualisation did show a strengthening and enduring influence over intraoperative performance over laparoscopic training. Finally, the results also highlighted that different visuospatial abilities play a role in different laparoscopic interventions. Visuospatial abilities were particularly associated with intraoperative performance in cholecystectomy, appendectomy and totally extra-peritoneal hernia interventions. Future researchers are encouraged to carefully consider these findings when wishing to evaluate visuospatial abilities in context of intraoperative performance.

The results of this study carry important implications for surgical education and future research on the topic. First, they seem to suggest that spatial visualisation may prove to be a valuable ability for residency evaluation and performance-based assessments. Such an approach coupled with technical evaluation could help educators and resident surgeons to self-monitor their learning and skill development progression. Second, the results do not support the notion of using visuospatial testing for purposes of residency selection, considering the extensive individual differences and experience-dependent nature of the abilities. As was observed in the scope of this study, visuospatial abilities are highly experience-dependent. Visuospatial testing would therefore result in an unfair selection of residents based on initial individual differences and not their potential in acquiring the necessary skills. Nevertheless, given the relationship between spatial visualisation and experience, spatial visualisation testing could be potentially used to guide selection for subspecialty training such as advanced minimally invasive fellowship programmes, but further research is required to investigate this further prior to its implementation. Third, considering the current understanding that spatial visualisation improves with laparoscopic training, the need for additional hand-on training outside the OR is further emphasis, particularly with the negative global impact of COVID-19 pandemic on surgical training [22]. As was demonstrated by Keehner [23], spatial visualisation was similarly associated with simulation-based laparoscopic performance. Additional lab-based training in conjunction with OR training could therefore prove to be a useful method for fast-tracking skill acquisition and competence attainment.

Future researchers are cautioned to closely consider the seeming malleable nature of abilities in the context of laparoscopic performance in the scope of their studies. As was demonstrated by the current findings, visuospatial abilities are highly experience-dependent. Special care must be taken when making inferences about the role of visuospatial ability in laparoscopy. Researchers are encouraged to more closely consider which abilities were measured, the characteristics of the subjects, their experience levels and the type of the intervention conducted when drawing inferences from their studies. Yet, this current study is also not without limitations and several factors ought to be considered when interpreting the results. As its norm in longitudinal studies, the study did encounter drop-out and missing data over the 27-month period. Five subjects dropped out of the study due to either unexpected relocation or resident surgeons leaving their respective clinics or rotating around departments in other hospitals. Respectively, the small and unequal sample size also influenced the results. For example, the GVVT did show a medium effect size (η2 = 0.5) with a clear trend towards significance (*p* = 0.06) when exploring differences between senior surgeons, residency surgeons and control subjects. Considering the clear pattern observed in the longitudinal analysis, it begs the question whether these group differences where simply concealed by the unequal sample size. Additionally, this study was highly impacted by the COVID-19 pandemic and cancellation of elective surgery between January and July 2020. This resulted in missing data in the last quarter of the study (month 24–27), mainly among residency surgeons.

## Conclusions

This longitudinal cohort study showed that visuospatial abilities associate with laparoscopic skills and improve with training. Spatial visualisation may be characteristic of laparoscopic expertise as it has clear and systematic association with competency development during laparoscopy residency training programme.

## Supplementary Information

Below is the link to the electronic supplementary material.

Supplementary file1 (DOCX 410 KB)
