# Supplementary material for: The development of visuospatial abilities and their impact on laparoscopic skill acquisition: a clinical longitudinal study
Source: Surg Endosc. 2022 May 31;36(12):8908–17. doi: 10.1007/s00464-022-09328-1 (PMC9154204; doi:10.1007/s00464-022-09328-1)

Appendix A.

1. The Perspective Taking/ Spatial Orientation Test (PTSOT) (adapted by Hegarty & Waller, 2004) measuring spatial orientation. The test depicts an array of 2D objects from which the subject is instructed to take a perspective from and draw a direction to another object within the array. The measure includes 12 items with a five-minute time limit.
2. A modified Guay’s Visualization of Views Test (GVVT) (adapted by Hegarty, Keehner, Khooshabeh & Montello, 2009) measuring spatial visualisation (i.e. 2D into 3D transformation). The test depicts a 3D figure inside a transparent cube seen from a 2D perspective with an additional illustration of the figure seen from a certain perspective (i.e. target). The subject is instructed to indicate the corner of the cube from which the illustrated target figure would be seen from. The test consists of 24 items with an eight-minute time limit.
3. A Mental Rotation Test (MRT-A) (Peters, Laeng, Latham, Jackson, Zaiyouna & Richardson, 1995) measuring mental rotation. The test depicts pairs of 3D figures rotated in various axes from which the subject is instructed to state which figures are identical and which are mirror figures. The measure comprises of 24 items with a six-minute time limit.

(4) The Pictorial Surface Orientation (PicSOr) (Gallagher, Cowie, Crothers, Jordan-Black & Satava, 2003) a computerised test measuring perceptual-motor skills. The test depicts a 3D figure from a 2D view viewed from various perspectives from which the subject manoeuvres a spinning arrowhead on that figure to the point they believe the arrow is perpendicular to the surface of the figure. The measure comprises of 35 items and has no time limit.

Appendix B. Graphical illustration of visuospatial trajectories for spatial orientation (PTSOT), mental rotation (MRT-A) and perceptual-motor skills


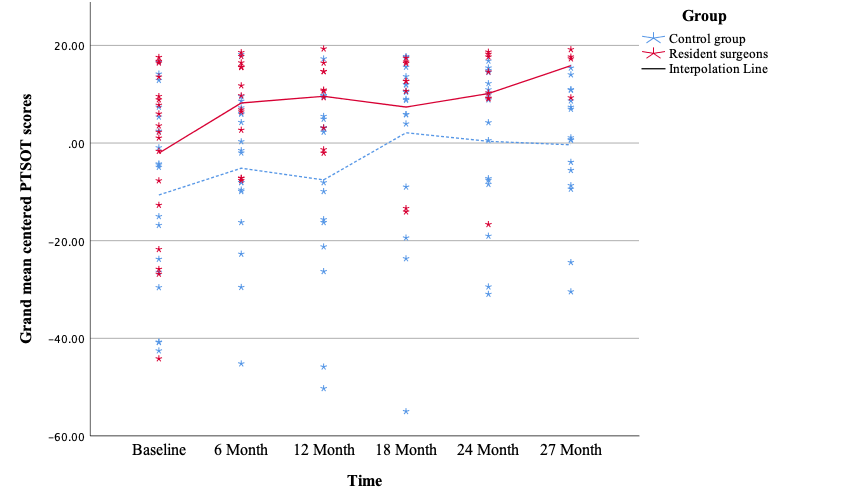

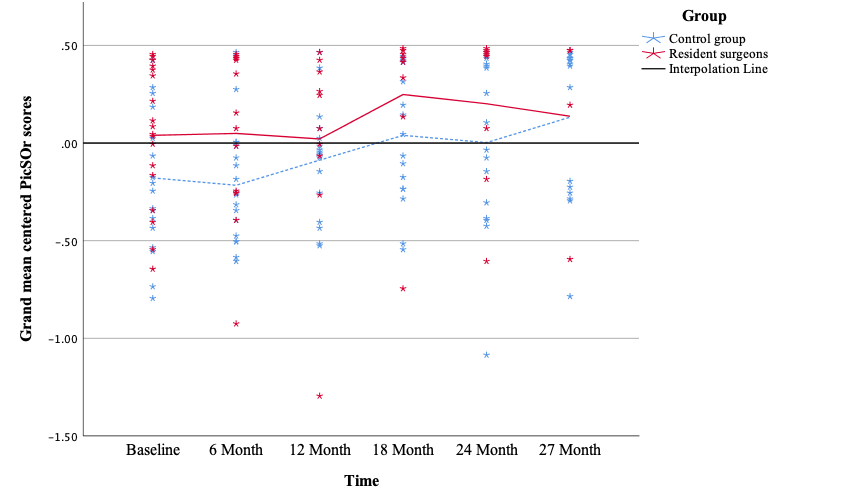

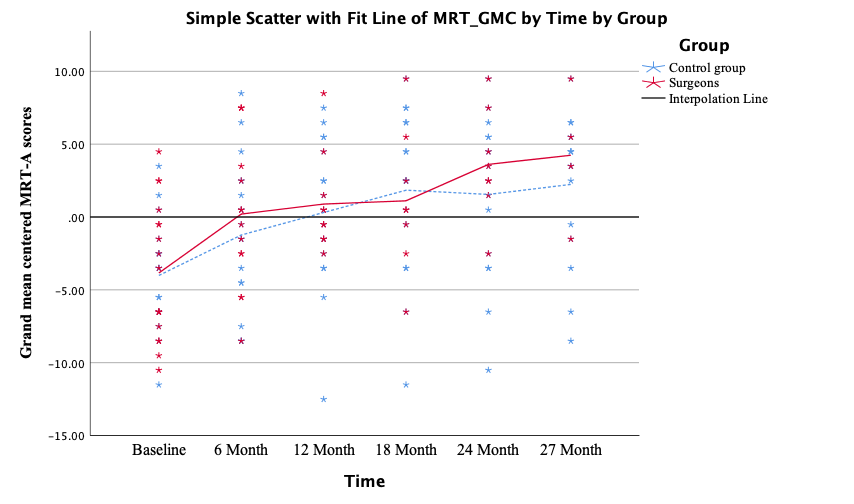

Supplement: Supplementary file 1 — Supplementary file1 (DOCX 410 KB) [file 464_2022_9328_MOESM1_ESM.docx]
